# Intra-Articular Injections Prior to Total Knee Arthroplasty Do Not Increase the Risk of Periprosthetic Joint Infection: A Prospective Cohort Study

**DOI:** 10.3390/antibiotics10030330

**Published:** 2021-03-21

**Authors:** Jérôme Grondin, Pierre Menu, Benoit Métayer, Vincent Crenn, Marc Dauty, Alban Fouasson-Chailloux

**Affiliations:** 1 CHU Nantes, Service de Médecine Physique et de Réadaptation Locomotrice, University Hospital of Nantes, 44093 Nantes, France; jerome.grondin@chu-nantes.fr (J.G.); pierre.menu@chu-nantes.fr (P.M.); marc.dauty@chu-nantes.fr (M.D.); 2 CHU Nantes, Service de Médecine du Sport, University Hospital of Nantes, 44093 Nantes, France; 3 INSERM UMR U1229/RMeS, Regenerative Medicine and Skeleton, Nantes University, 44000 Nantes, France; 4 IRMS, Institut Régional de Médecine du Sport, Hôpital Saint Jacques, 44093 Nantes, France; 5 CHU Nantes, Service de Rhumatologie, University Hospital of Nantes, 44093 Nantes, France; benoit.metayer@chu-nantes.fr; 6 CHU de Nantes, Clinique Chirurgicale Orthopédique et Traumatologique, Hôtel-Dieu, 44093 Nantes, France; vincent.crenn@chu-nantes.fr; 7 Physos, Inserm UMR 1238, Nantes University, 44000 Nantes, France

**Keywords:** knee, total knee arthroplasty, infection, intra-articular injection

## Abstract

Periprosthetic joint infections (PJI) occur in 0.5 to 2.8% of total knee arthroplasties (TKA) and expose them to an increase of morbidity and mortality. TKA are mainly performed after failure of non-surgical management of knee osteoarthritis, which frequently includes intra-articular injections of corticosteroids or hyaluronic acid. Concerning the potential impact of intra-articular injections on TKA infection, literature provides a low level of evidence because of the retrospective design of the studies and their contradictory results. In this prospective cohort study, we included patients after a total knee arthroplasty, at the time of their admission in a rehabilitation center, and we excluded patients with any prior knee surgery. 304 patients were included. Mean follow-up was 24.9 months, and incidence proportion of PJI was 2.6%. After multivariate logistic regression, male was the only significant risk factor of PJI (OR = 19.6; *p* = 0.006). The incidence of PJI did not differ between patients who received prior intra-articular injections and others, especially regarding injections in the last 6 months before surgery. The use of intra-articular injection remains a valid therapeutic option in the management of knee osteoarthritis, and a TKA could still be discussed.

## 1. Introduction

Periprosthetic Joint Infection (PJI) constitutes one of the most feared complications after total knee arthroplasties (TKA) [1]. PJI increases mortality, with a 71.7% overall survival five years after PJI diagnosis [2] and exposes them to the complications of challenging surgical and medical treatments [3,4,5]. It also reduces physical function and impairs quality of life [6,7]. Its incidence ranges from 0.5 to 2.8% according to the studies [8,9,10]. TKA is a frequent surgical procedure, increasing in number every year [11]. There is a great concern about prevention of PJI, and different recommendations have been published [12,13]. Yet, despite these recommendations, the rate of PJI apparently does not decrease over time [2].

TKA improves primary outcomes of knee osteoarthritis (KOA) such as pain and function [14], and is mainly performed after failure of medical treatment. Intra-articular injection remains an usual treatment of non-surgical KOA in the absence of absolute contraindications such as infectious arthritis and drug hypersensitivity [15], but guidelines are contradictory regarding its efficiency and safety [16,17]. During the procedure of intra-articular injections, a contamination of the joint may happen and potentially induce a PJI if an arthroplasty is secondarily performed [1]. In 2017, the Centers for Disease Control and Prevention (CDC) broached the topic, but the issue was considered unresolved, and no recommendation was made [13]. In clinical practice, intra-articular infiltrations of corticosteroids (CS) or hyaluronic acid (HA) are frequently performed [18], and around 30% of the patients who underwent TKA had previously had an intra-articular steroid injection [19]. In this context, many studies have been performed, but have provided a low level of evidence because of their retrospective design and contradictory results [10,19,20,21,22,23,24,25] (Supplementary Materials Table S1). Among them, three studies based on large databases have highlighted an increased risk of PJI if prior intra-articular injections had been performed in the few months preceding the surgery [10,19,20], but they were exposed to common limitations with large database studies. A few meta-analyses were performed on PJI after TKA or Total Hip Arthroplasty (THA) [18,26,27,28], also with contradictory results, and emphasizing the low level of evidence of available studies and the need for prospective trials.

Thus, we aimed to prospectively assess the impact of prior intra-articular injections on the occurrence of periprosthetic joint infection after TKA.

## 2. Results

**Figure 1:**
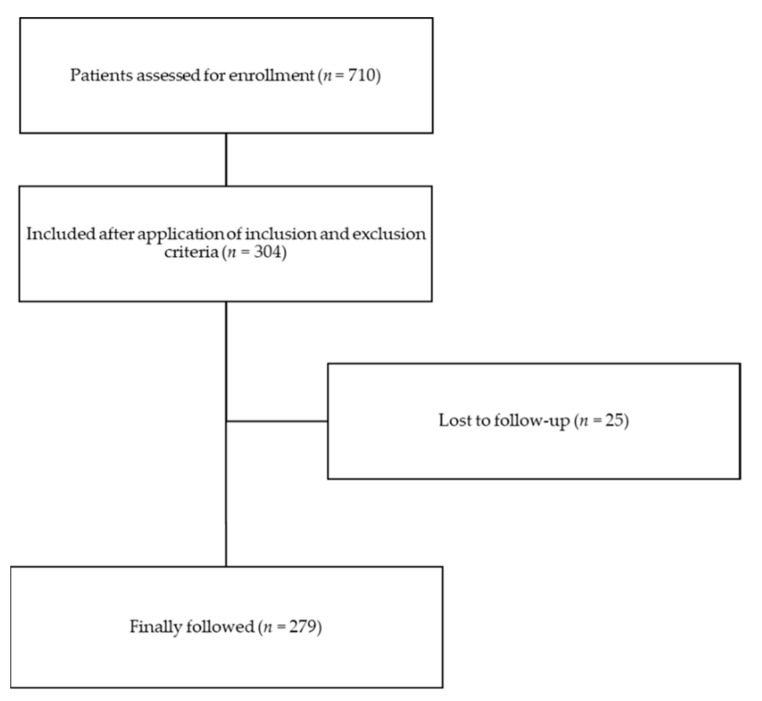
Flow-chart.

Between January 2016 and May 2019, 304 patients were included, and 279 (91.8%) eventually followed, while 25 patients (8.2%) were lost to follow-up (Figure 1). Mean follow-up was 24.9 months ± 3.8.

Most of the patients were females (72.4%; *n* = 220), and mean age was 71.8 years ± 8.9. Mean body mass index (BMI) was 30.9 kg/m^2^ ± 5.3 and 85.5% (*n* = 260) of the patients were overweight (BMI > 25) or obese (BMI > 30) at the time of the surgery (35.8% overweight (*n* = 109), 49.7% obese (*n* = 151)) (Table 1). Mean American Society of Anesthesiologists (ASA) score was 2.3 ± 0.6. Two patients were deceased 5 and 7 months after the arthroplasty (1 heart failure due to myocardial ischemia, and 1 cerebral stroke). 68.1% (*n* = 207) of the patients received infiltration before surgery, 48.8% (*n* = 101) of them with hyaluronic acid alone, 15.5% (*n* = 32) with corticosteroids, and 24.6% (*n* = 51) received both.

Table 2 summarizes the cases of PJI, mainly males (6 out of 8). Most of the infections (7/8) occurred in the first 6 weeks following arthroplasty and were caused by Staphylococcus aureus (6/8) or Staphylococcus capitis (1/8). The remaining case concerns a patient who initially received a surgery consisting of irrigation and debridement in a context of infectious endocarditis due to a persistent PJI, and a one-stage exchange was secondly performed. One patient died from myocardial ischemia 5 months after diagnosis of PJI. Other surgical and medical strategies performed were all considered successful, and no additional surgery was necessary.

The overall incidence of infection was 2.6% (8/304). Comparisons of incidence of PJI were completed with Fisher’s exact test depending on the “injection” status. Incidence was 2.1% (2/97) in patients without prior injection, and 2.9% (6/207) if any prior intra-articular injection had been performed, OR = 1.42 (CI 95% = 0.28–7.16; *p* = 0.67). It increased to 7.1% (3/42) if injection had been performed within 6 months before surgery, OR = 3.95, but without statistical significance (CI 95% 0.91–17.21; *p* = 0.08).

In univariate regression, the “sex” variable was the only one to be significantly associated with PJI, with an increased risk of infection in males. A trend was found concerning “injection < 6 months” with an OR of 3.46 (*p* = 0.09) (Table 3). Based on these findings, we have investigated potential differences between males and females that could explain the increased risk of PJI in males (Table 4). Therefore, we have highlighted significant differences between the two groups: Smoking, diabetes, alcoholism, and ASA score were significantly higher in males than in females. 

Multivariate logistic regressions were performed considering differences between males and females. In the total population, only sex was significantly associated with occurrence of infection (OR = 19.6; CI95%: 2.4–164; *p* = 0.006). Knowing existing differences between males and females in our population, we performed multivariate logistic regressions analyzing these 2 groups separately: No factor was significantly associated with PJI occurrence. 

## 3. Discussion

In this study, the risk of PJI did not significantly increase between patients who had previously received knee infiltration and patients who had not [OR = 1.42 (CI 95% = 0.28–7.16; *p* = 0.67)]. Many studies have been performed concerning the safety of intra-articular infiltrations in the pre-operative period, with a retrospective design and conflicting results [10,19,20,21,22,23,24,25]. Four of these studies did not bring out significant associations. However, 3 studies based on large retrospective databases suggested an increased risk of PJI in patients who had received an infiltration in the 3 months preceding surgery [10,19] or even in the preceding 7 months [20]. These findings explain why we compared the occurrence of infection between patients who had received an infiltration in the 6 months preceding surgery to the others. There was no significant difference, but a trend toward an increased risk in patients who had received an infiltration in the 6 months preceding surgery (OR = 3.95; CI 95% 0.91–17.21; *p* = 0.08). As discussed below, this trend requires further investigation with larger cohorts in prospective studies. Thus, special attention should be paid to the benefit/risk assessment of a knee infiltration if a surgery is to be scheduled in the next months.

In previous retrospective studies based on large prospective databases, confounding factors such as male sex, BMI, tobacco smoking, prior surgery, and inflammatory arthritis may have been involved in the significant association reported between PJI and prior intra-articular injections [10,19,20]. As recommended in previous systematic reviews [28], we clearly excluded patients with major risk factors of infection: Any prior surgery or septic arthritis of the knee, history of rheumatoid arthritis or hemophilia, and immunosuppressive or immunomodulatory drugs. We also adjusted the results on potential confounding factors previously reported: Male sex, age < 60 years, BMI > 25 kg/m^2^, diabetes, previous or current tobacco smoking, ASA ≥ 3 [29,30,31,32]. In our cohort, male sex was the unique risk factor associated with infection. Smoking, diabetes, alcoholism, and ASA score were significantly higher for males than females. In multivariate logistic regression, excluding male sex, no factor was significantly associated with PJI. Further analysis focusing on male population did not bring out significant results, especially regarding prior intra-articular injection in the 6 months preceding surgery.

Thus, despite conflicting evidence regarding the potential association between PJI and pre-operative joint injection, some pathophysiological hypotheses were suggested: An infectious risk due to the prolonged immunosuppressive effect of glucocorticoids injected [10,24,33], or direct inoculation from the infiltration procedure due to insufficient sterile precautions [10,24]. To investigate these hypotheses, we planned a 24 month-follow-up. Indeed, the first 2 years are the greatest risk period and represent 60 to 70% of PJI [11,34], and studies with shorter follow-ups have reported lower incidence of infection [8]. Furthermore, early (<3 months) and delayed infections (between 3 and 24 months after surgery) are often exogenous, early infections caused by more virulent organisms than delayed ones; whereas late-onset infections (>24 months) are frequently due to hematogenous infection [1,11], except in cases of very indolent infections due to very low-virulent bacteria [11]. These pathophysiological hypotheses are unlikely to explain late-onset hematogenous infections, which is why we did not follow patients for more than 2 years. In our cohort, every infection occurred within 6 months after surgery, most of them within 6 weeks, consistent with the hypotheses of exogenous pathogenesis.

We selected a telephone follow-up, for it produces higher response rates than postal survey or mail/internet surveys [35,36,37]. However, the telephone mode brings more positive responses to subjective items than other modes [37], but this bias does not apply in our case, since the interview was closed-ended to detect the occurrence of PJI. A memorization bias may be suggested in principle, but patients would unlikely forget a Periprosthetic Joint Infection with its devastating consequences, revision surgeries, and extended antibiotic therapy.

This study has limitations. Indeed, our cohort was formed with patients admitted in a Physical and Rehabilitation Medicine Hospital, which are usually different from those discharged home directly after surgery: They are usually older, with a higher BMI, and are more frequently females [38]. In our cohort as well, patients were mostly females (72.4%) with a mean BMI of 30.9. Periprosthetic joint infections are usually estimated between 1 and 2% after TKA [30], but may range over 2% [9,10]. In this study, the incidence proportion was 2.6%, which seems consistent with literature knowing that we included more fragile patients. The main limitation was the size of the cohort (around 300 patients), which may have reduced its ability to detect a statistically significant association between intra-articular infiltrations and PJI. However, at the beginning of the study, we calculated that 276 patients were required to detect a doubling of the incidence of infection. Therefore, we included 304 patients and eventually followed 279 of them, but our initial projection may be challenged. The number of patients needed to improve the power and allowing recommendations depends on the incidence of PJI in the population, on the difference in incidence proportion that we aimed to detect, and on the pre-specified power (usually 80%). Further prospective studies should be performed in larger cohorts to clearly establish the safety of intra-articular injections before total knee arthroplasty, in order to improve sensitivity and power. Yet, a single institution is unlikely to sustain such a study. A multicentric study proves to be necessary [28], but exposes us to specific bias of multicentric designs, such as unrecognized heterogeneity across centers [39].

Another limitation is the diagnosis and classification of PJI which are challenging and not consensual [40]. In this study, we used the definition of the International Consensus Meeting on Periprosthetic Joint Infection, and every case fulfilled at least one of the two major criteria of PJI [41,42]. Different classifications exist, mainly based on timing of clinical presentation leading to different surgical strategies [40,43], and therefore decreasing comparability between studies.

Finally, the telephone follow-up might have failed to detect some PJI signs, especially in case of indolent infections due to low-virulent bacteria, which usually provide few clinical manifestations. Indeed, clinical, radiological, and biological parameters may have been more sensitive.

## 4. Materials and Methods

### 4.1. Participants

Patients were included in the few days following the arthroplasty (2 to 7 days), at the time of their admission in the rehabilitation center. Surgery was performed in the University Hospital of Nantes or in other clinics of Nantes’ region, France. Inclusion criteria were: Age > 18 years old, patients hospitalized for rehabilitation after TKA. Exclusion criteria were: Any prior ipsilateral knee surgery, any prior infectious arthritis of the knee, history of rheumatoid arthritis or hemophilia, and immunosuppressive or immunomodulatory drugs.

At the time of the inclusion, we systematically collected the following data: Age, sex, weight, height, BMI, diabetes, tobacco smoking, alcoholism, ASA Score, other significant medical and surgical antecedents, date and place of surgery, prior intra-articular infiltration of the knee: Number, date, and type of medication injected.

### 4.2. Outcome

The primary outcome was the incidence of PJI. Every case of infection was reviewed and defined as a PJI if it fulfilled the definition provided by the International Consensus Meeting on Periprosthetic Joint Infection (at least one of the two major criteria: Two positive growths of the same organism using standard culture methods, or sinus tract with evidence of communication to the joint or visualization of the prosthesis) [41,42]. First, we compared incidences of infection between patients who had received prior intra-articular injections and others, and then we focused on patients who had had an intra-articular injection in the 6 months preceding surgery.

### 4.3. Follow-Up

Follow-up was performed at 24 months after surgery. A phone call was performed, and occurrences of an infection or an additional surgery were checked based on following questions: “Do you feel any persistent knee pain, erythema and oedema?”, “Have you noticed any wound drainage?”, “Has a diagnosis of prosthetic infection or any infection of your knee been established? “Have you got any additional surgery?”. If any of these occurred, medical, surgical, and bacteriological reports were gathered. If a patient was not able to answer these questions, his general practitioner was called.

### 4.4. Statistical Analyses

Statistical analyses were performed using software SPSS 23.0 IBM Corp, Armonk, NY, USA. Comparisons of incidence proportions of PJI were performed with a Fisher’s exact test. Logistic regressions were performed with PJI as dichotomous dependent variable, and independent variables were sex, age, BMI, ASA, diabetes, smoking, alcoholism, prior infiltration (Yes/No), infiltration < 6 months (Yes/No). First, we analyzed the association between dependent and independent variables in univariate regression, and then we performed a multivariate logistic regression with forward selection (Wald). We compared demographic characteristics between males and females using *t*-test. *p* < 0.05 was considered significant. To evaluate the number of subjects required, we defined a power of 80%, an alpha risk of 5%, a theoretical incidence of PJI of 2.8% [10], and aimed to detect a doubling of the incidence. We calculated that 276 patients were required.

### 4.5. Ethics

This research was conducted in our institution from January 2016 to June 2019. Due to the non-interventional nature of the study, no ethics committee was necessary at the time of the beginning of the study. Yet, necessary processes were performed with the “Direction de la Recherche Clinique” (DRC) of the University Hospital of Nantes, France, and the “Commission Nationale de l’Informatique et des Libertés” (CNIL); the study was registered under the number RC16_0039. The database was anonymized, and all the patients provided their verbal consent and got an information document.

## 5. Conclusions

This study showed no evidence of the causality of prior intra-articular injections in Periprosthetic Joint Infection occurrence, even in the 6 months preceding surgery. In clinical practice, wise use of intra-articular injection remains a valid therapeutic option in the management of knee osteoarthritis, and a total knee replacement could still be discussed.

## Figures and Tables

**Table 1:** Demographic characteristics.

Characteristics	Patients (*n* = 304)
Mean age, years ± SD	71.8 ± 8.9
[min–max]	[31–91]
Sex:	
-Female, *n* (%)	220 (72.4)
-Male, *n* (%)	84 (27.6)
Mean weight, kg ± SD	82.0 ± 16.3
[min–max]	[46–149]
Mean height, cm ± SD	162.9 ± 9.3
[min–max]	[136–190]
Mean BMI, kg/m^2^ ± SD	30.9 ± 5.3
[min–max]	[19.4–47.6]
Diabetes mellitus:	
-Type 1, *n* (%)	7 (2.3)
-Type 2, *n* (%)	43 (14.1)
-None, *n* (%)	254 (83.6)
Smoking:	
-Active, *n* (%)	14 (4.6)
-Cessation, *n* (%)	49 (16.1)
-None, *n* (%)	241 (79.3)
Alcoholism:	
-Active, *n* (%)	23 (7.5)
-Cessation, *n* (%)	5 (1.7)
-None, *n* (%)	276 (90.8)
Mean ASA Score, mean ± SD	2.3 ± 0.6
[min–max]	[1–4]
Prior IA injection, *n* (%):	207 (68.1)
-CS	32 (15.5)
-HA	101 (48.8)
-CS+HA	51 (24.6)
-Unknown	23 (11.1)
No prior IA injection, *n* (%)	97 (31.9)

SD: Standard-deviation; BMI: Body mass index; ASA: American society of anesthesiologists; IA: Intra-articular; CS: Corticosteroids; HA: Hyaluronic acid.

**Table 2:** Cases of periprosthetic joint infections.

Patients	Age Years	Sex F/M	BMI	Diabetes	Smoking	Alcoholism	ASA	Infiltrations			Delay Surgery-PJI	Surgery	Bacteria	Antibiotic Therapy	
								DTS	Medication	*n*				Type	Duration
1	73	M	32.3	No	No	Yes	1	14 m	CS	1	11 d	Debridement, implant retention, replacement of exchangeable components	Methi-S Staph. aureus	Levofloxacin + Rifampicin	12 w
2	64	F	33.3	Type 2	No	No	3	-	-	0	4 w	One-Stage Arthroplasty Exchange	Methi-S Staph. aureus	Levofloxacin + Rifampicin	12 w
3	88	M	20.4	No	Former smoker	No	2	1 m	HA	9	5 w	One-Stage Arthroplasty Exchange	Methi-S Staph. aureus	Levofloxacin + Rifampicin	12 w
4	78	F	37.5	No	No	No	3	-	-	0	5 w	Debridement, implant retention, replacement of exchangeable components	Methi-S Staph. aureus	Levofloxacin + Clindamycin	12 w
5	85	M	28.7	No	No	No	3	4 m	CS	3	4 w	Debridement, implant retention, replacement of exchangeable components	Methi-S Staph. aureus	Levofloxacin + Rifampicin	12 w
6	53	M	26.9	No	No	No	2	12 m	HA	unknown	12 d	Debridement, implant retention, replacement of exchangeable components	Staph. capitis	Levofloxacin + Rifampicin	8 w
7	77	M	24.4	No	Former smoker	No	3	10 m	CS + HA	7	5 m	Initially irrigation and debridment, then 4 months later 1-Stage Arthroplasty Exchange	Strep. oralis	Moxifloxacin + Amoxicillin after lavage; Moxifloxacin + Clindamycin after 1-stage exchange	12 w
8	74	M	29.4	No	Current smoker	No	3	4 m	CS + HA	7	14 d	Debridement, implant retention, replacement of exchangeable components	Methi-R Staph. aureus	Cotrimoxazole + fusidic acid, then Clindamycin + fusidic acid (renal insufficiency)	12 w

F: Female; M: Male; BMI: Body mass index; ASA: American society of anesthesiologists; DTS: Delay to surgery; m: Months; d: Days; w: Weeks; CS: Corticosteroids; HA: Hyaluronic acid; PJI: Periprosthetic Joint Infections; Methi-S: Methicillin sensitive; Methi-R: Methicillin resistant, Staph.: Staphylococcus; Strep.: Streptococcus.

**Table 3:** Univariate logistic regression according to patients’ characteristics.

Independent Variables	Odds-Ratio	CI 95%	*p*
Age	1.03	0.94–1.12	0.48
Sex	0.05	0.006–0.41	0.005
BMI	0.93	0.8–1.08	0.35
Smoking	2.36	0.54–10.1	0.24
Diabetes mellitus	0.72	0.08–5.98	0.76
Alcoholism	1.77	0.2–15.1	0.59
ASA	2.12	0.6–7.43	0.23
Injection < 6 months	3.46	0.79–15	0.09

CI: Confidence interval; BMI: Body mass index; ASA: American society of anesthesiologists.

**Table 4:** Comparison between males and females.

Characteristics	Males *n* = 84	Females *n* = 220	*p*
Mean age, years ± SD	70.5 ± 8.8	72.3 ± 8.9	0.12 ^a^
Mean BMI, kg/m^2^ ± SD	30.6 ± 5.2	30.9 ± 5.4	0.71 ^a^
Smoking (Active or cessation), *n*	35	28	0.001 ^b^
Diabetes mellitus, *n*	21	29	0.01 ^b^
Active alcoholism, *n*	19	4	0.0001 ^b^
ASA ≥ 3, *n*	37	60	0.01 ^b^
Injection < 6 months, *n*	12	30	0.88 ^b^

^a^*t*-test; ^b^ χ^2^ -test. SD: Standard-deviation; BMI: Body mass index; ASA: American society of anesthesiologists.

## Data Availability

The datasets generated and/or analyzed during the current study are available from the corresponding author on reasonable request.

## Supplementary Materials

The following are available online at https://www.mdpi.com/2079-6382/10/3/330/s1, Table S1: Literature review of the impact of intraarticular injection before surgery on the occurrence of infection.
