# Supplementary material for: Intra-Articular Injections Prior to Total Knee Arthroplasty Do Not Increase the Risk of Periprosthetic Joint Infection: A Prospective Cohort Study
Source: Antibiotics (Basel). 2021 Mar 21;10(3):330. doi: 10.3390/antibiotics10030330 (PMC8004162; doi:10.3390/antibiotics10030330)
Supplement: Supplementary file 1 [file antibiotics-10-00330-s001.pdf]

antibiotics

MDPI

Supplementary Material

Table S1: Literature review of the impact of intraarticular injection before surgery on the occurrence of infection.

Author	Study Design	Patients, n	Results
Papavasiliou et al, 2006	Retrospective matched cohort study - 54 patients with intra-articular corticosteroids injection and 90 patients without	144	Significant association between administration of corticosteroids in the 11 months before surgery and occurrence of deep infection
Joshy et al, 2006	Retrospective matched cohort study - study group with 32 patients with confirmed deep infection, control group with 32 patients with no evidence of infection	64	No significant difference in number of patients who received intra-articular steroid injection between the groups
Horne et al, 2008	Retrospective matched cohort study - study group with 38 patients with infection, and control group with 352 patients without infection	390	No significant difference with intra-articular injection of steroids
Desai et al, 2009	Retrospective matched cohort study - 90 patients with intra-articular steroid injection prior to TKA and 180 patients without	270	No significant difference in incidence of infection
Amine et al, 2015	Retrospective - 783 patients with intra-articular injection prior to TKA and 845 without	1 628	No significant difference in incidence of infection
Cancienne et al, 2015	Retrospective, based on a national US database – 5313 patients with TKA within 3 months after knee injection, 8919 between 3 to 6 months, 8008 between 6 to 12 months, 13650 without knee injection	35 890	Significant increase of infection in patients who received intra-articular steroids injection > 3 months prior to TKA compared to patients without injection

Bedard et al, 2017	Retrospective, based on large database - 29 603 patients with intra-articular injection prior to TKA, 54 081 without	83 684	Significant increase of infection in patients who received intra-articular injection compared to patients without injection. Incidence of infection was higher in the injection cohort out to a duration of 6 months between injection and TKA
Richardson et al, 2019	Retrospective, based on national insurance database - 3249 patients who received hyaluronic acid, 16656 who received corticosteroids	58 337	Significantly increased risk of PJI infection in patients who received corticosteroids or hyaluronic acid injections < 3 months before TKA
